# Wood-decaying fungi found in Ghana: A rich source of new anti-infective compounds

**DOI:** 10.12688/aasopenres.12957.1

**Published:** 2019-06-07

**Authors:** Samuel Yaw Aboagye, Vincent Amarh, Paul A. Lartey, Patrick Kobina Arthur

**Affiliations:** 1 West African Center for Cell Biology of Infectious Pathogens, Department of Biochemistry, Cell and Molecular Biology, University of Ghana, Legon-Accra, P. O. Box LG54, Ghana; 2 LaGray Chemical Company, Nsawam, Ghana

**Keywords:** Wood decaying fungi, bioactive compounds, antimicrobial compounds, and infectious diseases

## Abstract

**Background:** Discovery of bioactive natural products are instrumental for development of novel antibiotics. The discovery and development of natural products such as penicillin represented a major milestone in the treatment of bacterial infections. Currently, many antibiotics have lost their relevance in clinics due to the emergence of drug-resistant microbial pathogens. Hence, there is the need for continuous search of new compounds endowed with potent antimicrobial activity.

**Methods:** In this study, wood-decaying fungi (WDF) from Ghana were explored for their potential as sources of novel antimicrobial compounds with intent of expanding the effort into a drug discovery programme in the near future. Extracts from cultures of 54 morphologically distinct WDF isolates were analyzed for the presence of antimicrobial agents.

**Results:** The extracts from 40 out of the 54 WDF isolates exhibited significant antimicrobial activity against either *Staphylococcus aureus*, *Escherichia coli* or *Candida albicans. *Fractionation of these bioactive extracts, followed by bioassay of the organic fractions obtained, indicate that extracts exhibiting antimicrobial activity against more than one of the three test organisms could be attributed to the presence of different bioactive compounds. Analysis of the composition of the extracts revealed that terpenes were predominant.

**Conclusions:** This study suggests that a significant proportion of WDF in Ghana produce antimicrobial compounds which could be potential sources of novel anti-infective agents and support the plans of developing a drug discovery programme in Ghana based on the fermentation of WDF.

## Introduction

Several plants and fungi have served as sources of many drugs that are used in clinics ^1, 2^. A high proportion of these drugs originated from fungal sources. Secondary metabolites from fungi are known to exhibit diverse biological activities, and therefore constitute a good resource for the discovery and development of novel bioactive compounds ^3^. The fact that fungal biodiversity is underexplored, especially in Africa, enhances the expectation that many new and diverse bioactive molecules can be discovered from these sources to serve as potent chemotherapeutic agents ^4, 5^. Novel drug candidates are required for treatment of infections caused by microbial pathogens, especially drug-resistant pathogens ^6, 7^. A typical example is the multi-drug resistant strains of *Mycobacterium tuberculosis*, for which treatment by antibiotic chemotherapy is proving to be very difficult. While several pathogens continue to evolve, the rate of antibiotic discovery has stalled, with many pharmaceutical companies closing their antibiotic discovery programs over the last 50 years ^6^.

A proportion of microbial pathogens that afflict mammals may possibly infect fungal species, since they are both eukaryotes with related cellular metabolism. Unlike mammals, fungi have a higher tendency to produce relevant chemical compounds for combating infectious pathogens. Therefore, exploring fungal secondary metabolites to identify suitable candidates which can be developed as novel antibiotics is not only timely but also urgent. Thus, our global public health settings can benefit immensely from the defense strategies used by fungi against pathogenic microorganisms such as *Escherichia coli* and *Staphylococcus aureus* ^8^.

Wood-decaying fungi (WDF) belong to the phylum Basidiomycetes; they constitute the major group of fungi responsible for degradation of organic matter ^9, 10^. Fungal metabolites, such as penicillin G, retapamulin and lentinan, have been used either as precursors or lead compounds for the development of pharmaceutical products. Penicillin is the pioneer of the antibiotic era of modern medicine used to control infectious diseases ^11^. Interestingly, while the majority of fungal metabolites have been used as antibiotics, Lentinan, a polysaccharide isolated from *Lentinus edodes*, has been commercialized for clinical use as an anti-cancer drug ^12^. Retapamulin is one of the recent antibiotics isolated from fungi and is currently used in clinics; it is a derivative of pleuromutilin isolated from *Clitopilus* sp. ^13– 16^. Fungal fermentation is also easily scalable creating the possibility of large-scale production systems once future effort leads to the discovery of high value products.

This study investigated the potential of indigenous WDF found in Ghana to produce antimicrobial compounds. This objective was achieved by collecting diverse morphologically distinct WDF from different locations in Ghana. A high proportion of these collection of indigenous WDF produced antimicrobial agents. This observation provides a strong indication that the immense fungal biodiversity can be a renewable resource of potent and novel antimicrobial agents, thereby forming the strong basis for a drug discovery programme to contribute to global health concerns.

## Methods

### Collection of WDF isolates

Images were acquired for each fungus that was found growing on dead wood. Each fungus was collected into a tightly capped plastic container and stored, for the long-term, at room temperature. For this study, the WDF were collected from several suburbs in Ghana, including the main campus of the University of Ghana, Aburi forest, Lashibi, Ridge Hospital and Cape Coast. A total of 54 morphologically distinct WDF were collected. Most of these WDF were found growing under humid conditions over the period between October 2009 and April 2010. All the WDF that were collected were given unique codes; each fungus was coded using a combination of letters and numbers (A1–A9, B1–B9, C1–C9, D1–D9, E1–E9 and F1–F9). The location and date of harvest were also documented; these information were necessary for identification of seasonal variation of fungal isolates, as well as preference for specific habitats ^17^.

### Preparation and inoculation of potato dextrose broth (PDB) with WDF

Irish potatoes were used for preparing liquid broth in 500-ml culture bottles, under strict asceptic conditions; 80 g of potatoes was used for preparing 200 ml of broth in each culture bottle. The 200 ml potato broth was supplemented with 4 g of dextrose, under asceptic conditions.

For each WDF isolate, about 5 g of fruiting body was thoroughly washed and added to 200 ml of freshly prepared PDB under aseptic conditions. These freshly inoculated PDB were incubated at room temperature for 48 days with daily swirling to enhance aeration.

### Determination of dextrose clearance by WDF in PDB culture

An aliquot of 100 µl of each WDF culture was subjected to centrifugation at 5000 *g* for 5 minutes. The supernatants obtained were used for the determination of dextrose concentration using the On-Call Plus Glucometer, according to the manufacturer’s instruction.

### Extraction of metabolites from the WDF cultures

An equal volume of ethyl acetate (200 ml) was added to each WDF culture and was followed by vigorous shaking to ensure complete extraction of metabolites into the organic phase. After separation, the organic phase from each WDF culture was evaporated to dryness to obtain crude extracts. The extract from each WDF culture was reconstituted in 200 µl absolute methanol and stored at -4°C.

### Analysis of WDF extracts by thin-layer chromatography (TLC)

TLC was performed using silica gel 60 coated with the F _254_ fluorescent indicator on aluminum plates (Sigma Aldrich). An aliquot of 5 µl of each WDF extract was spotted approximately 1 cm from the bottom of the TLC plate. The spots were dried and developed in a saturated TLC glass tank using the solvent system, ethyl acetate: acetonitrile: petroleum ether (7:2:1), as mobile phase. During TLC analysis, the mobile phase migrated from the bottom to the top of the silica gel in approximately 15 minutes at room temperature. The developed TLC plates were air-dried, and the separated bands visualized under visible and UV light (254 nm and 365 nm). Subsequently, the TLC plates were sprayed with anisaldehyde reagent (135 ml of absolute ethanol, 5 ml of concentrated sulphuric acid, 1.56 ml of glacial acetic acid and 3.7 ml of *p*-anisaldehyde), heated at 100°C for 5 minutes and examined under visible light.

### Fractionation of WDF extracts by Sephadex LH-20 chromatography

The WDF extracts were fractionated by sephadex LH-20 chromatography; particle size of the sephadex was 5 µm (Pharmacia). A concentration of 25% (w/v) LH-20 slurry was prepared using methanol as solvent. The chromatography column was made in-house with a length of 16 cm and a bed volume of 80 ml. An aliquot of 1 ml WDF extract was loaded onto the column and was initially allowed to flow slowly into the matrix using very small volume of solvent. To ensure good separation of extracts, the flow rate was kept at approximately 1 ml/min. Three fractions of 10 ml were collected as void volumes, followed by ten fractions for each WDF extract. At the end of the tenth collection, the column was washed with the eluting solvent (methanol) to remove any remaining metabolite retained by the column. The ten fractions were concentrated using a rotary evaporator and the samples were recovered in methanol and stored at -20°C.

### Antimicrobial assay of WDF extracts

An aliquot of 40 µl of each WDF extract was added to sterile Whatmann paper discs (6 mm in diameter) under aseptic conditions. The test organisms, *Staphylococcus aureus* ATCC.2, *Escherichia coli* NMIMR.3, and *Candida albicans* ATCC.2 were grown in nutrient broth and standardized to a concentration of 3 × 10 ^7^cells/ml using a BaSO _4_ turbidity equivalent to a 0.5 McFarland standard. Sterile cotton swaps were used to uniformly streak the adjusted bacterial suspension onto agar plates prepared from nutrient broth. The air-dried paper discs impregnated with the WDF extracts were uniformly placed on the inoculated nutrient agar plates. Streptomycin, kanamycin and ciclopirox olamine (products from La Gray Chemical Company) were used as positive controls at a concentration of 30 μg per disc for the three test organisms. Sterile paper discs impregnated with either ethyl acetate or methanol were air-dried and used as negative controls. Each plate was examined for the presence of zones of inhibition after 18 hours of incubation at 37°C. All bioassays were performed in duplicates. The diameter of the zones of inhibition were measured from three different points of the circle and the average was calculated.

## Results

### Metabolism of WDF isolates in PDB

Foams were detected within the broth of the WDF cultures from the 3 ^rd^ to the 18 ^th^ day of incubation at room temperature. Foaming of the cultures might be indicative of the production of gases by these WDF during growth in PDB; these gases could represent by-products of cellular metabolism. It has been reported that the transition from primary to secondary metabolism is marked by the depletion of nutrients in the culture medium ^18^. This study investigated the time point at which the dextrose was completely metabolized by the WDF during culturing in PDB; 2 out of the 54 WDF isolates were selected and used for this assay. The initial dextrose concentration in the freshly inoculated PDB was 550 mmol/L and decreased drastically to 40 mmol/L by the 3 ^rd^ day of incubation. Dextrose levels were not detected in the PDB on the 8 ^th^ day of incubation. This observation could suggest that transition to secondary metabolism occurred prior to the 8 ^th^ day of incubation of the fungal cultures and might represent the onset of production of antimicrobial compounds. Unprocessed results of dextrose metabolism assays, in addition to all other raw results, are available as *Underlying data* ^19^.

### Antimicrobial activities of extracts from WDF cultures

After 48 days of incubation of the WDF cultures, the metabolites produced were extracted and air-dried. The WDF isolates with codes C7, E5, F2, F4 and F6 produced the highest yield of extract (200 mg) while the lowest yield (25 mg) was obtained from the WDF isolate designated B3. A significant number of the extracts were either yellowish or creamy in color.

**Figure 1.**
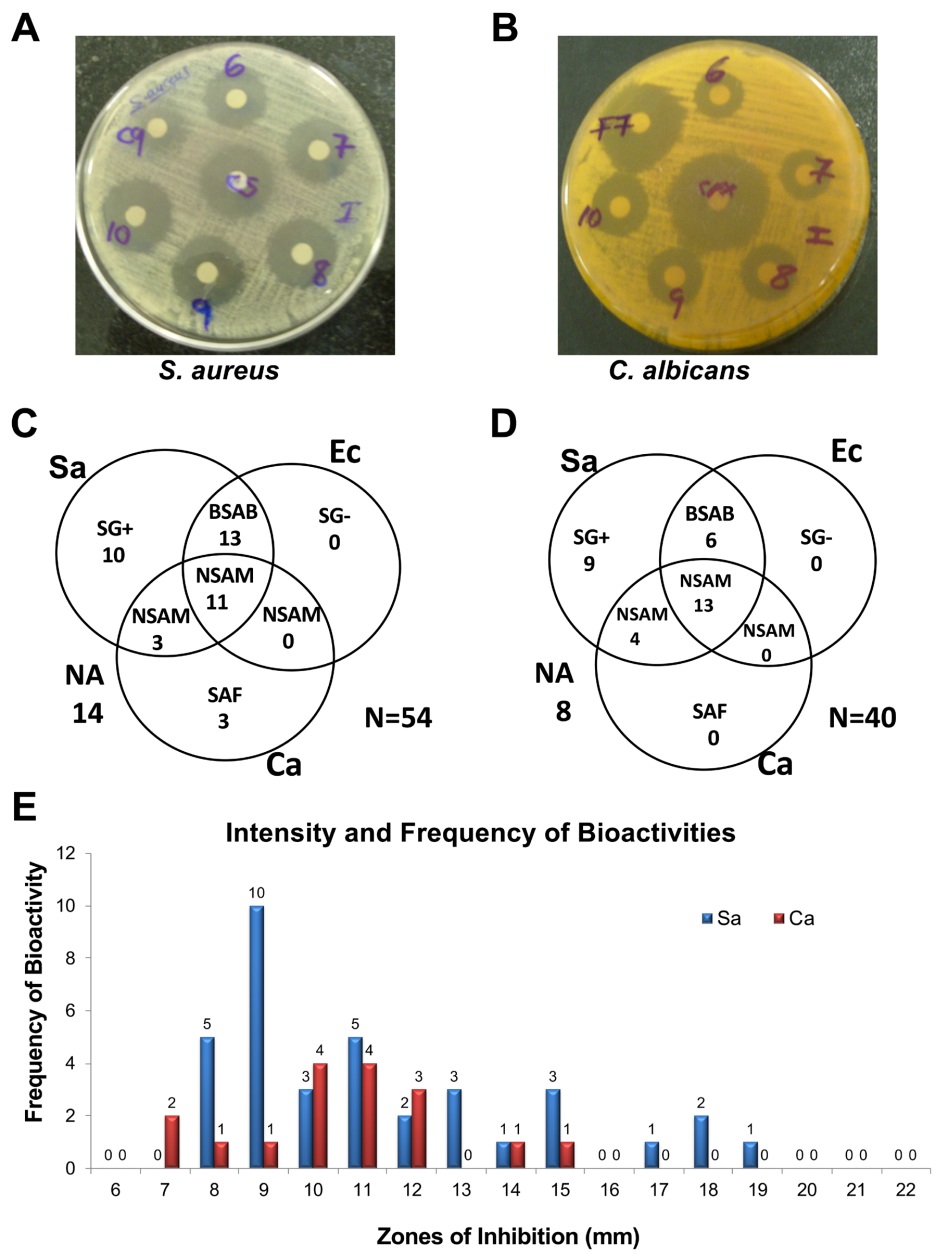
Primary and confirmatory screening for antimicrobial activities produced by WDF against *S. aureus*, *E. coli* and *C. albicans*. Examples of bioassay plates of wood-decaying fungi (WDF) extracts showing growth inhibition zones for *S. aureus* ( **A**) and *C. albicans* ( **B**) with streptomycin and cyclopirox olamine control antibiotics, respectively. Venn diagram showing the antimicrobial activities detected in the primary screen for 54 WDF extracts ( **C**) and confirmatory screen for 40 WDF extracts ( **D**). The frequency and potency of antimicrobial activity against *S. aureus* and *C. albicans* for the primary screening of the 54 WDF extracts ( **E**). NSAM, non-selective antimicrobial activity; BSAB, broad-spectrum antimicrobial; SG+, selective antibacterial activity against Gram-positive *S. aureus*; SAF, selective antifungal activity against *C. albicans*; SG-, selective antibacterial activity against Gram-negative *E. coli*.

In order to establish whether the WDF isolates were capable of producing antimicrobial agents, the extract from each of the 54 fungal cultures were tested against *S. aureus*, *E. coli* and *C. albicans* using the disc diffusion assay ( Figure 1A, B). The assay revealed that 40 out of the 54 extracts exhibited antimicrobial activity against at least one of the test organisms. Out of these 40 bioactive extracts, 11 extracts exhibited non-selective antimicrobial activity (NSAM) towards all the three test organisms, while 13 extracts showed antibacterial activity towards both *S. aureus* and *E. coli*. Additionally, 10 extracts exhibited selective antibacterial activity against only the gram-positive (SG+) *S. aureus*, while three other extracts showed selective antifungal activity (SAF) towards *C. albicans*. Notably, none of the 54 extracts exhibited selective antibacterial activity against only the gram-negative (SG-) *E. coli* ( Figure 1C).

In order to validate the production of antimicrobial compounds from the 40 WDF isolates, they were re-cultured in 1-litre PDB. Re-culturing of each of these 40 WDF was performed in 1 litre of PDB to ensure that sufficient extracts were obtained for the subsequent fractionation and biochemical assays. Foaming of the fungal cultures was detected from the 3 ^rd^ to the 21 ^st^ day of incubation at room temperature. The bioassay data of the extracts from these 40 WDF cultures showed that 8 of the extracts did not retain their antimicrobial activity against neither of the three test organisms. Moreover, none of the 40 extracts exhibited selective antifungal activity against *C. albicans*. Despite these observations, 80% of the 40 extracts showed antimicrobial activity against at least one of the test organisms ( Figure 1D), thereby validating the production of antimicrobial compounds from these indigenous WDF. Interestingly, four of the extracts showed higher antibacterial activity against *S. aureus*, relative to streptomycin (10 μg) which had a zone of inhibition of 16 mm ( Figure 1E).

### Time course for production of antimicrobial compounds from WDF cultures

**Figure 2.**
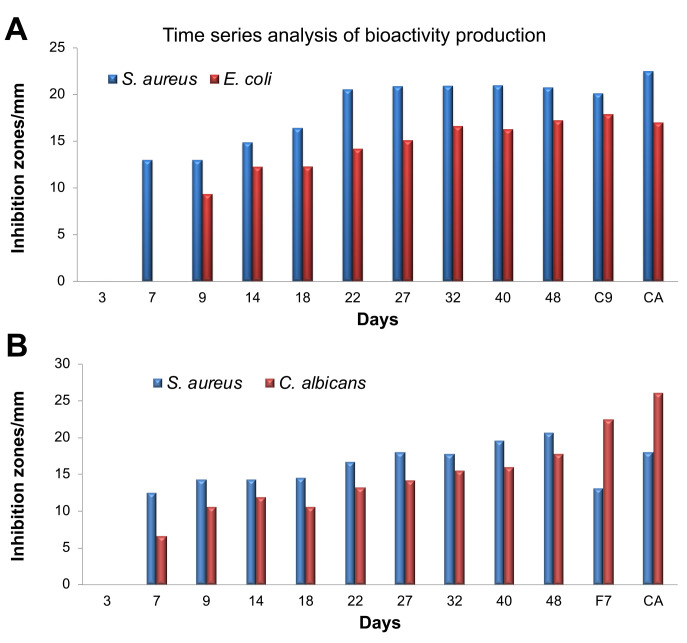
Time series analysis for the production of antimicrobial compounds by wood-decaying fungi C9 and F7 against *S. aureus* and *E. coli* ( **A**) and *S. aureus* and *C. albicans* ( **B**). Control antibiotics (CA): *S. aureus*, Streptomycin; *E. coli*, Kanamycin; *C. albicans*, Cyclopirox olamine. Antimicrobial activity of the extracts from the indicated time points was measured using the disc-diffusion assay, along with control antibiotics and reference samples of both C9 and E7.

Our earlier observation that WDF completely depleted dextrose from PDB by the 8 ^th^ day of incubation of the fungal cultures led us to hypothesize that these WDF began production of secondary metabolites by day 8. The two WDF isolates that were used for the dextrose depletion assay were chosen to investigate the time course of secondary metabolite production by the WDF cultures. This investigation was relevant for determining the duration required by each WDF to attain maximum production of secondary metabolites in their respective cultures. The two selected WDF (C9 and F7) were each cultured separately in PDB for durations of 3, 7, 9, 14, 18, 22, 24, 32, 40, and 48 days. Ethyl acetate was added to these cultures at the indicated time point to terminate fungal growth and to extract the secondary metabolites produced. No distinct and consistent band pattern were observed for the TLC profiles of these extracts. However, when these extracts were used for bioassays, it was observed that the C9 cultures produced antimicrobial compounds against *S. aureus* starting from the 7 ^th^ day and it attained maximum productivity of these compounds on the 22 ^nd^ day of culturing. These C9 extracts also exhibited antibacterial activity against *E. coli* from the 9 ^th^ day of culturing and maximum production of antimicrobial compounds was attained on day 32. In comparison, *S. aureus* was found to be more susceptible to extracts from the C9 cultures than *E. coli* over the entire sampling period ( Figure 2A).

Extracts from the F7 cultures exhibited antimicrobial activity against both *C. albicans* and *S. aureus* from the 7 ^th^ day and increased steadily until the 48 ^th^ day of culturing. The zones of inhibition observed for *S. aureus* were usually bigger than those for *C. albicans* ( Figure 2B). These observations indicate that the complete depletion of dextrose from the fungal cultures by day 8 is a good indicator of initiation of secondary metabolite production, including antimicrobial compounds.

### Fractionation of WDF extracts exhibiting multiple antimicrobial activities

The data from the antimicrobial assays revealed that a significant number of the WDF extracts exhibited antimicrobial activity against more than one of the three test organisms. In order to ascertain whether this observation was due to the presence of multiple bioactive compounds in each of these extracts, six WDF extracts were selected and fractionated using a sephadex LH-20 column. Extracts of WDF with codes A4, B6, B7, E2, E9 and F3 were selected because they exhibited antimicrobial activity against two of the test organisms. An aliquot of each of these extracts was fractionated via sephadex LH-20 column chromatography and the fractions obtained were tested for antimicrobial activity against *S. aureus*, *C. albicans* and *E. coli.*

**Figure 3.**
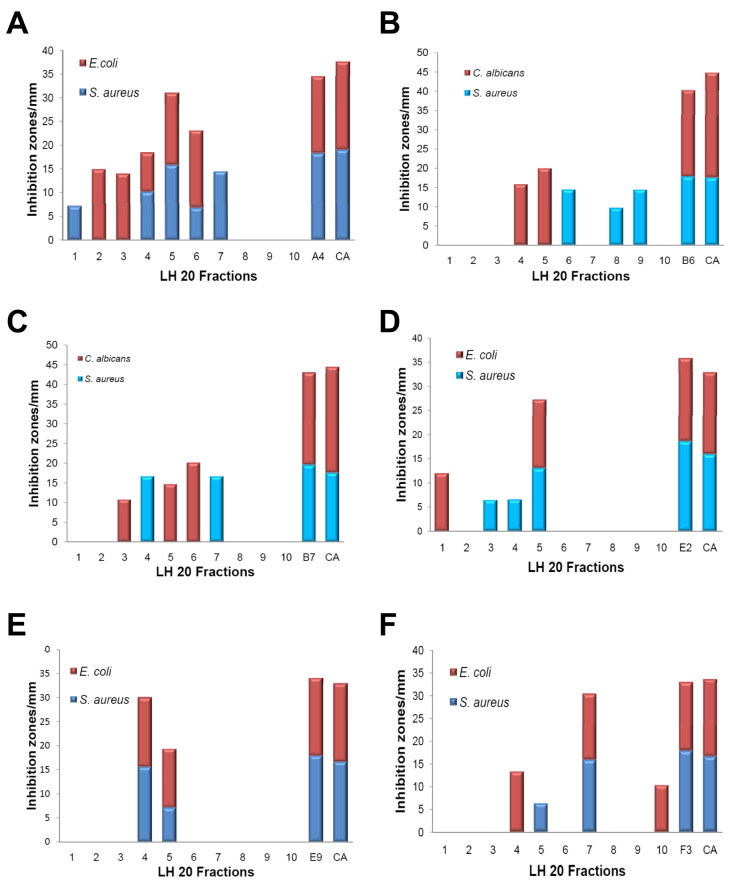
Analysis of the fractionated wood-decaying fungi (WDF) extracts exhibiting multiple antimicrobial activities. Prior to the bioassay, six selected WDF extracts (A4, B6, B7, E2, E9 and F3) were fractionated by sephadex LH-20 chromatography. The antimicrobial activities of the fractions obtained were measured using the disc diffusion assay with control antibiotics and original unfractionated extracts. The plot of zone of inhibitions for A4 ( **A**), B6 ( **B**), B7 ( **C**), E2 ( **D**), E9 ( **E**) and F3 ( **F**). Control antibiotics (CA): *S. aureus*, Streptomycin; *E. coli*, Kanamycin; *C. albicans*, Cyclopirox olamine.

The A4 extract had five fractions that exhibited antimicrobial activity against *S. aureus*. The zones of inhibition that were recorded for two of these fractions (fractions 2 and 5; Figure 3A) were relatively high, suggesting that either a high amount of a single bioactive compound was present in these fractions or multiple bioactive compounds were present in each fraction. Similarly, five of the fractions from the A4 extract also exhibited antimicrobial activity against *E. coli*. Interestingly, three of these fractions (fractions 4, 5 and 6) exhibited broad-spectrum activity against both *S. aureus* and *E. coli* ( Figure 3A). Fractions from the B6 extract, which were tested for bioactivity against *S. aureus* and *C. albicans*, had three fractions showing antibacterial activities against *S. aureus* while two other fractions exhibited antifungal activity against *C. albicans* ( Figure 3B).

For the B7 extract, antibacterial activity against *S. aureus* was detected in two out of the ten fractions whilst three other fractions showed antifungal activity against *C. albicans* ( Figure 3C). One fraction from the E2 extract exhibited antibacterial activities against both *S. aureus* and *E. coli* ( Figure 3D). Moreover, selective antibacterial activities against *S. aureus* was detected in two of the fractions from the E2 extract whilst only one fraction was active *E. coli* alone ( Figure 3D). E9 was the only extract with two bioactive fractions, both of which showed broad-spectrum antibacterial activity against *S. aureus* and *E. coli* ( Figure 3E). For the F3 extract, three fractions showed selective antibacterial activity against *E. coli* while two fractions were active against *S. aureus* ( Figure 3F). Collectively, these observations may suggest that the broad-spectrum antimicrobial activities exhibited by the extracts selected for this assay, were predominantly due to the presence of different antimicrobial compounds in these extracts. Nonetheless, the bioactive LH 20 fractions from these six extracts would require additional purification procedures in order to confirm the presence of different antimicrobial compounds within each of these extracts.

### Terpenes are the predominant compounds produced by the collection of WDF

**Figure 4.**
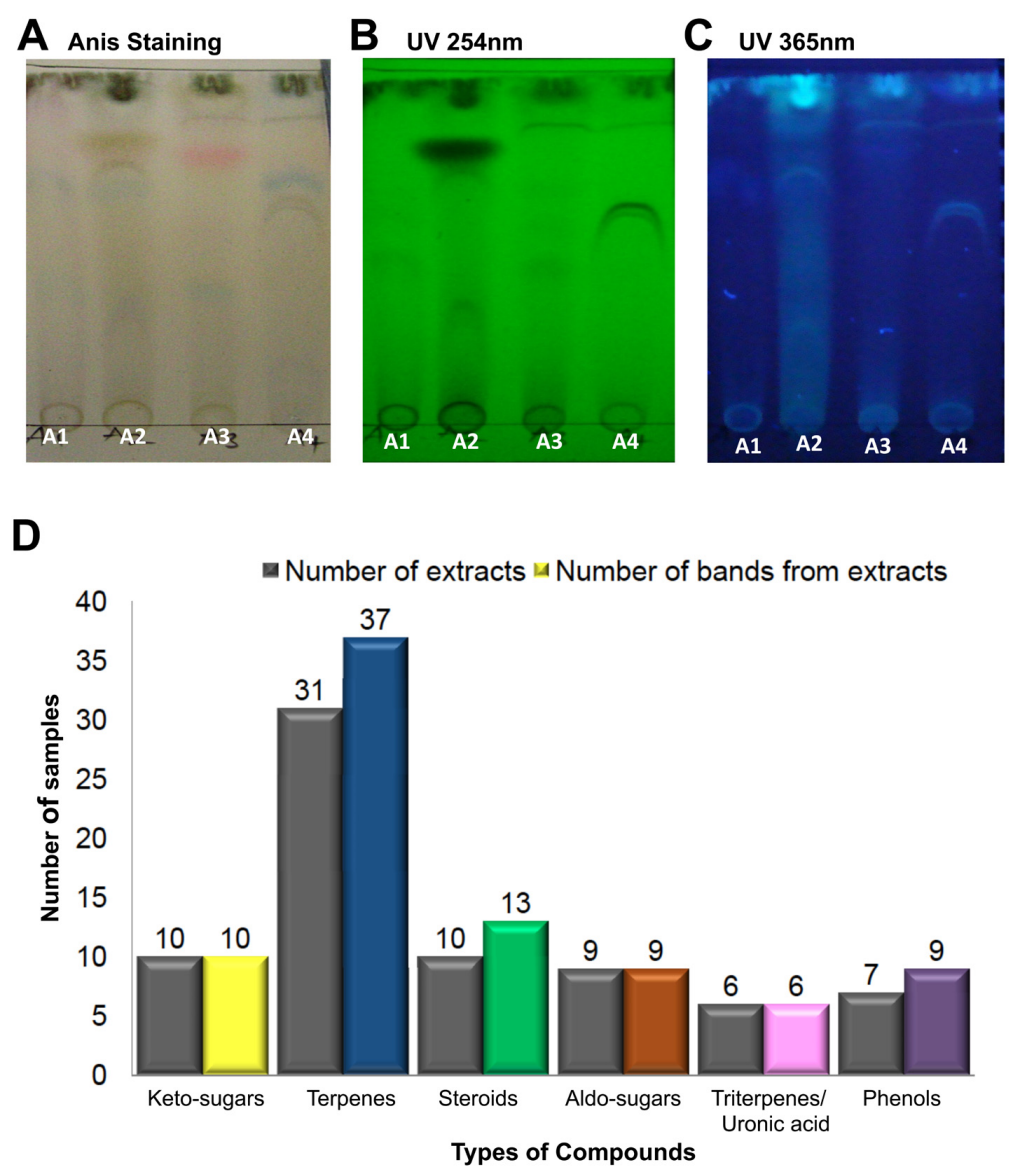
Diversity and distribution of compounds on the anisaldehyde-stained thin-layer chromatography (TLC) plates. The wood-decaying fungi (WDF) extracts A1, A2, A3 and A4 were run on TLC plates using the following solvent system as mobile phase; ethyl acetate: acetonitrile: petroleum ether (7:2:1). The TLC plates were visualized under UV and sprayed with anisaldehyde reagent in visible light. Examples of TLC plates showing the output of the various detection systems used in the analysis: ( **A**) Anisaldehyde staining ( **B**) UV 254 nm, ( **C**) UV 365 nm. ( **D**) The number of WDF extracts producing the indicated compounds (first bar) and the total number of individual colored bands produced by the WDF (second bar); the compound types are inferred from the corresponding colors.

TLC analysis was performed in order to study the diversity of compounds in the fungal extracts ( Figure 4). Following separation of the extracts on the TLC plates, a detection system consisting of the native band colors, UV light and anisaldehyde staining were used for visualization of the unique pattern of compounds produced by the fungal cultures. The anisaldehyde staining of TLC plates was very useful for determining the types of compound produced by the fungal cultures. Each extract from the 54 WDF cultures produced several bands on the TLC plate, with different profiles and Rf values, which suggest that each of the WDF isolates were distinct from each other. After staining with anisaldehyde, the colors of the bands on the TLC plates were indicative of the presence of compounds such as keto-sugars (yellow), terpenes (blue), aldo-sugars (brown), phenols (purple), steroids (green) and uronic acids (pink) ^20^. TLC bands corresponding to terpenes (blue bands) were observed in 31 out of the 54 WDF extracts; a few of these 31 extracts showed more than one distinct blue band. Thus, a total of 37 blue bands were detected from these 31 fungal extracts. Uronic acids and phenolic bands were found in 6 and 7 WDF extracts, respectively. Additionally, steroids and keto-sugars were detected in 10 WDF extracts, either exclusively or together with other compound types. Correlation analysis revealed that most WDF extracts exhibiting antimicrobial activities also produced terpenes. Hence, it is likely that a significant proportion of the antimicrobial compounds could be terpenes.

## Discussion

This study explored the potential of indigenous WDF as sources of new antimicrobial compounds. A high proportion of these indigenous WDF exhibited either antibacterial or antifungal activities, demonstrating that these fungi are sources of antimicrobial compounds. Even though this study did not perform subsequent purification of the bioactive LH 20 fractions to obtain the relevant antimicrobial compounds in their pure form, it can be postulated that novel antimicrobial compounds can be obtained from these indigenous WDF. Moreover, the use of only a small collection of WDF isolates for this study indicates that the great biodiversity of WDF in Ghana represents potential sources of new compound structures which could be developed as novel drugs against microbial pathogens. The drug discovery programme initiated through this precursor study will conduct large scale fermentation to allow the exploitation of the vast diversity of bioactive compounds detected in this project.

It has previously been reported that the majority of bioactive compounds isolated from the fungus *Corynespora* sp. were polyketides ^21^. The predominant compounds detected in the present study were terpenes. The strong correlation between the occurrence of these terpenes and antimicrobial activity suggests a high probability of isolating terpenes as antimicrobial compounds. The fact that terpenes are yet to be explored for development of novel antibiotics underpins the relevance of this study for drug discovery ^1^. The presence of other types of compounds in the bioactive WDF extracts could imply that the antimicrobial efficiency of some of these terpenes could be dependent on the presence of these other compounds. Thus, the synergy of terpenes with other compounds such as steroids, glycosides and phenols could be exploited for development of combination chemotherapy against both drug susceptible and multidrug resistant microbial pathogens.

The antimicrobial activity of the WDF extracts illustrate the relevance of fungal secondary metabolism to drug discovery. The preliminary data provided by this study also shows that the complete depletion of readily-available nutrients (such as dextrose) from the culture medium leads to initiation of fungal secondary metabolism. We recommend further studies that would investigate optimal conditions for culturing these indigenous WDF in order to boost immediate accumulation of antimicrobial agents following initiation of fungal secondary metabolism. Ultimately, it is intended that these efforts would contribute to efficient isolation of several new drug candidates to enhance the global fight against infectious diseases.

## Conclusion

This study has demonstrated that the great biodiversity of WDF in Ghana represents a valuable resource for discovery of antimicrobial agents, many of which might be novel to the drug discovery industries.

## Data availability

Open Science Framework: Wood Decaying Fungi (WDF) as Anti-infective Source Study. https://doi.org/10.17605/OSF.IO/H5GP9 ^19^.

The file “WDF and anti-infectives study Raw Data_Additional file.zip” contains the data underlying the results of this study, including antimicrobial activity at each time point, dextrose concentrations, zones of inhibition, images of plates and antimicrobial activity following repeated culture.

Data are available under the terms of the Creative Commons Zero "No rights reserved" data waiver (CC0 1.0 Public domain dedication).
