# Wood-decaying fungi found in Southern Ghana: A potential source of new anti-infective compounds

**DOI:** 10.12688/aasopenres.12957.2

**Published:** 2019-08-27

**Authors:** Samuel Yaw Aboagye, Vincent Amarh, Paul A. Lartey, Patrick Kobina Arthur

**Affiliations:** 1 West African Center for Cell Biology of Infectious Pathogens, Department of Biochemistry, Cell and Molecular Biology, University of Ghana, Legon-Accra, P. O. Box LG54, Ghana; 2 LaGray Chemical Company, Nsawam, Ghana

**Keywords:** Wood decaying fungi, bioactive compounds, antimicrobial compounds, and infectious diseases

## Abstract

**Background:** Discovery of bioactive natural products are instrumental for development of novel antibiotics. The discovery and development of natural products such as penicillin represented a major milestone in the treatment of bacterial infections. Currently, many antibiotics have lost their relevance in clinics due to the emergence of drug-resistant microbial pathogens. Hence, there is the need for continuous search of new compounds endowed with potent antimicrobial activity. In this study, wood-decaying fungi (WDF) from Southern Ghana were explored for their potential as sources of novel antimicrobial compounds with intent of expanding the effort into a drug discovery programme in the near future.

**Methods:** A total of 54 WDF isolates were fermented in potato dextrose broth and the secondary metabolites obtained were analyzed for the presence of antimicrobial agents using the disc diffusion assay. Chromatography techniques were used for preliminary analysis of the chemical composition of the extracts and for fractionation of the extracts that showed antimicrobial activity.

**Results:** The extracts from 40 out of the 54 WDF isolates exhibited significant antimicrobial activity against either *Staphylococcus aureus*, *Escherichia coli* or *Candida albicans. *Fractionation of these bioactive extracts, followed by bioassay of the organic fractions obtained, indicate that extracts exhibiting antimicrobial activity against more than one of the three test organisms could be attributed to the presence of different bioactive compounds. Analysis of the composition of the extracts revealed that terpenes were predominant.

**Conclusions:** This study suggests that a significant proportion of WDF in Southern Ghana produce antimicrobial compounds which could be potential sources of novel anti-infective agents and support the plans of developing a drug discovery programme in Ghana based on the fermentation of WDF.

## Introduction

Several plants and fungi have served as sources of many drugs that are used in clinics ^1, 2^. Secondary metabolites from fungi are known to exhibit diverse biological activities, and therefore constitute a good resource for the discovery and development of novel bioactive compounds ^3^. The fact that fungal biodiversity is underexplored, especially in Africa, enhances the expectation that many new and diverse bioactive molecules can be discovered from these sources to serve as potent chemotherapeutic agents ^4, 5^. Novel drug candidates are required for treatment of infections caused by microbial pathogens, especially drug-resistant pathogens ^6, 7^. A typical example is the multi-drug resistant strains of *Mycobacterium tuberculosis*, for which treatment by antibiotic chemotherapy is proving to be very difficult. While several pathogens continue to evolve, the rate of antibiotic discovery has stalled, with many pharmaceutical companies closing their antibiotic discovery programs over the last 50 years ^6^.

A proportion of microbial pathogens that afflict mammals may possibly infect fungal species, since they are both eukaryotes with related cellular metabolism. Unlike mammals, fungi have a higher tendency to produce relevant chemical compounds for combating infectious pathogens. Therefore, exploring fungal secondary metabolites to identify suitable candidates which can be developed as novel antibiotics is not only timely but also urgent. Thus, our global public health settings can benefit immensely from the defense strategies used by fungi against pathogenic microorganisms such as *Escherichia coli* and *Staphylococcus aureus* ^8^.

Wood-decaying fungi (WDF) belong to the phylum Basidiomycetes; they constitute the major group of fungi responsible for degradation of organic matter ^9, 10^. Fungal metabolites, such as penicillin G, retapamulin and lentinan, have been used either as precursors or lead compounds for the development of pharmaceutical products. Penicillin is the pioneer of the antibiotic era of modern medicine used to control infectious diseases ^11^. Interestingly, while the majority of fungal metabolites have been used as antibiotics, Lentinan, a polysaccharide isolated from *Lentinus edodes*, has been commercialized for clinical use as an anti-cancer drug ^12^. Retapamulin is one of the recent antibiotics isolated from fungi and is currently used in clinics; it is a derivative of pleuromutilin isolated from *Clitopilus* sp. ^13– 16^. Fungal fermentation is also easily scalable creating the possibility of large-scale production systems once future effort leads to the discovery of high value products.

This study investigated the potential of indigenous WDF found in Ghana to produce antimicrobial compounds. This objective was achieved by collecting diverse WDF from different locations in Southern Ghana. The rate of utilization of glucose by these WDF and duration for incubation of WDF cultures for optimal production of antimicrobial secondary metabolites were also investigated. A high proportion of the collection of indigenous WDF produced antimicrobial agents. This observation provides a strong indication that the immense fungal biodiversity can be a renewable resource of potent and novel antimicrobial agents, thereby forming the strong basis for a drug discovery programme to address global health concerns.

## Methods

### Collection of WDF isolates

**Figure 1.**
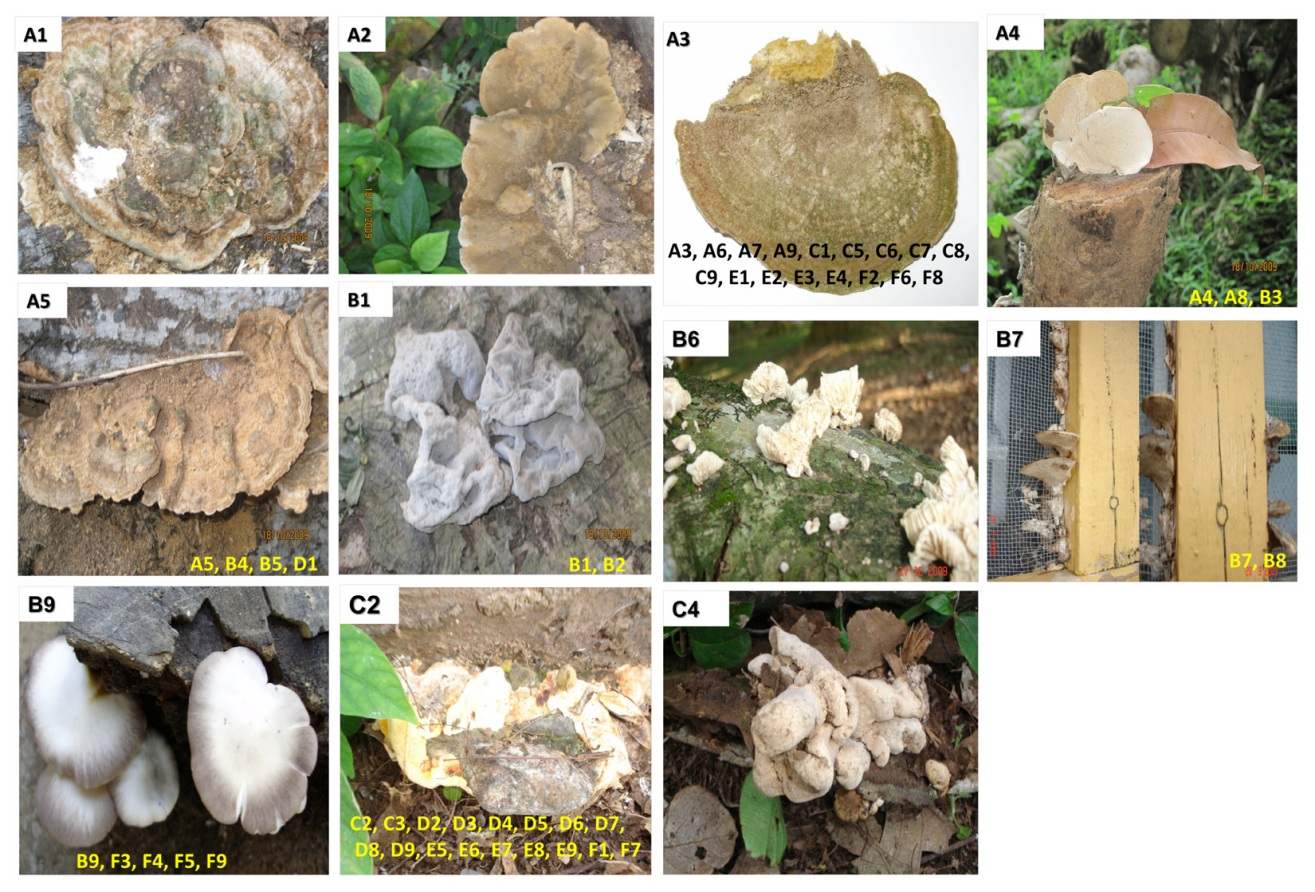
Representative images of the 54 WDF isolates. For each of the 11 images, the code of the fungus is indicated at the top left corner. The codes of other fungi which had fairly similar morphology to the images shown are also indicated at the bottom of the images.

A total of 54 WDF were collected from several suburbs in Southern Ghana, including the main campus of the University of Ghana, Aburi forest, Lashibi, Ridge Hospital and Cape Coast. These WDF were found growing under humid conditions over the period between October 2009 and April 2010. All the 54 fungi were found growing on dead wood; images of each fungus attached to the wood were acquired prior to harvesting the fungus into a tightly capped plastic container. The fungi were stored in the plastic container at room temperature for the long-term. All the WDF that were collected were given unique codes; each fungus was coded using a combination of letters and numbers (A1–A9, B1–B9, C1–C9, D1–D9, E1–E9 and F1–F9). The location and date of harvest were also documented; these information were necessary for identification of seasonal variation of fungal isolates, as well as preference for specific habitats ^17^. Even though the WDF were harvested from different locations, some of the isolates had fairly similar physical morphology and were assigned to a category. Thus, a total of 11 categories were obtained from the 54 WDF isolates based on similarities in morphology ( Figure 1).

### Preparation and inoculation of potato dextrose broth (PDB) with WDF

PDB was used for culturing each of the 54 WDF isolates in 500-ml bottles. The broth was prepared from Irish potatoes under strict asceptic conditions; 80 g of potatoes was used for preparing 200 ml of broth in each culture bottle. The 200 ml potato broth was supplemented with 4 g of dextrose, under asceptic conditions.

For each WDF isolate, approximately 5 g of fruiting body was thoroughly washed and added to the 200 ml of freshly prepared PDB under aseptic conditions. These freshly inoculated PDB were incubated at room temperature for 48 days with daily swirling to enhance aeration. For the validation of production of antimicrobial compounds by these fungi, 40 out of the 54 WDF isolates were re-cultured in 1-litre PDB using 2-litre culture bottles; these 40 isolates were selected because they produced antimicrobial agents from the initial 200ml cultures. Re-culturing of each of these isolates was performed in 1 litre of PDB to ensure that sufficient extracts were obtained for the subsequent fractionation and biochemical assays.

### Determination of dextrose clearance and production of antimicrobial metabolites from WDF cultures

The complete utilization of dextrose from WDF cultures was used as an indicator for initiation of production of secondary metabolites in these cultures. Dextrose clearance assay was performed by aliquoting 100 μl of culture from the PDB inoculated with C9 and F7 on days 0, 3, 7, 9, 14, 18, 22, 27, 32, 40 and 48. The aliquots obtained from both cultures on the indicated days were stored at -20 °C. These aliquots were thawed and span at 5000 *g* for 5 minutes and the supernatants obtained were used for determination of dextrose concentration using the On-Call Plus Glucometer, according to the manufacturer’s instruction.

The two WDF isolates (C9 and F7) were also used for inoculation of replicates (ten for each WDF isolate) of 50 ml PDB in order to obtain insight on the time course for production of secondary metabolite by these WDF cultures. The ten replicates of inoculated PDB for each of the two selected WDF (C9 and F7) were incubated at room temperature for the following durations: 3, 7, 9, 14, 18, 22, 24, 32, 40, and 48 days. Equal volume of ethyl acetate was added to the two cultures (representing each WDF isolate) at the indicated time points to terminate fungal growth and to extract the secondary metabolites that were produced.

### Extraction of metabolites from the WDF cultures

The secondary metabolites produced by each WDF culture, at the indicated days of incubation, were extracted using equal volume of ethyl acetate. After addition of ethyl acetate, the cultures were shaken vigorously to ensure complete extraction of metabolites into the organic phase. After separation of the organic phase from the aqueous cultures, the former was decanted and evaporated to dryness to obtain crude extracts. The extract from each WDF culture was reconstituted in absolute methanol (50 µl, 200 µl and 1000 µl of methanol for extracts from 50 ml, 200 ml and 1 litre cultures, respectively) and stored at 4°C.

### Analysis of WDF extracts by thin-layer chromatography (TLC)

Extracts from the 54 WDF cultures were analyzed by TLC to obtain preliminary insights on the diversity of compounds produced by each fungus. The TLC was performed using silica gel 60 coated with the F _254_ fluorescent indicator on aluminum plates (Sigma Aldrich). An aliquot of 5 µl of each WDF extract was spotted approximately 1 cm from the bottom of the TLC plate. The spots were dried and developed in a saturated TLC glass tank using the solvent system, ethyl acetate: acetonitrile: petroleum ether (7:2:1), as mobile phase. During TLC analysis, the mobile phase migrated from the bottom to the top of the silica gel in approximately 15 minutes at room temperature. The developed TLC plates were air-dried, and the separated bands were visualized under visible and UV light (254 nm and 365 nm). The visualization of TLC plates under UV-light was useful for determining the presence of UV-light absorbing compounds, which is predictive of the presence of compounds containing a conjugated pi system. The TLC plates were also sprayed with anisaldehyde reagent (135 ml of absolute ethanol, 5 ml of concentrated sulphuric acid, 1.56 ml of glacial acetic acid and 3.7 ml of *p*-anisaldehyde), heated at 100°C for 5 minutes and examined under visible light The colours that develop on the anisaldehyde-stained TLC plates are indicative of the presence of specific functional groups: keto-sugars (yellow), terpenes (blue), aldo-sugars (brown), phenols (purple), steroids (green) and uronic acids (pink) ^18^.

### Fractionation of WDF extracts by Sephadex LH-20 chromatography

A total of 6 out of the 54 WDF extracts were fractionated by sephadex LH-20 chromatography; these extracts (A4, B6, B7, E2, E9, and F3) were chosen because they exhibited high antimicrobial activity against two of the test organisms in both the primary and secondary screening (disc diffusion) assays. A concentration of 25% (w/v) sephadex LH-20 slurry was prepared using methanol as solvent; particle size of the sephadex was 5 µm (Pharmacia). The chromatography column was made in-house with a length of 16 cm and a bed volume of 80 ml. An aliquot of 850 μl WDF extract was loaded onto the column and was initially allowed to flow slowly into the matrix using very small volume of solvent. To ensure good separation of extracts, the flow rate was kept at approximately 1 ml/min. Three fractions of 10 ml were collected as void volumes, followed by ten fractions for each WDF extract. At the end of the tenth collection, the column was washed with the eluting solvent (methanol) to remove any remaining metabolite retained by the column. The ten fractions were concentrated using a rotary evaporator and the samples were recovered in methanol and stored at -20°C.

### Antimicrobial assay of WDF extracts

Extracts from all the WDF cultures were tested for the presence of antimicrobial agents using the disc diffusion assay. All fractions obtained from the sephadex LH-20 chromatography were also tested for the presence of antimicrobial compounds using the disc diffusion assay. An aliquot of 40 µl of each WDF extract (or 5mg/ml for the fractions) was added to sterile Whatmann paper discs (6 mm in diameter) under aseptic conditions. The test organisms, *Staphylococcus aureus* ATCC.2, *Escherichia coli* NMIMR.3, and *Candida albicans* ATCC.2 were grown in nutrient broth and standardized to a concentration of 3 × 10 ^7^cells/ml using a BaSO _4_ turbidity equivalent to a 0.5 McFarland standard. Sterile cotton swaps were used to uniformly streak the adjusted bacterial suspension onto agar plates prepared from nutrient broth. The air-dried paper discs impregnated with the WDF extracts were uniformly placed on the inoculated nutrient agar plates. Streptomycin, kanamycin and ciclopirox olamine (products from La Gray Chemical Company) were used as positive controls at a concentration of 30 μg per disc for the three test organisms. Sterile paper discs impregnated with either ethyl acetate or methanol were air-dried and used as negative controls. Each plate was examined for the presence of zones of inhibition after 18 hours of incubation at 37°C; plates inoculated with *C. albicans* were incubated at 30 °C for 12 hours. All bioassays were performed in duplicates. The diameter of the zones of inhibition were measured from three different points of the circle and the average was calculated.

## Results

### Metabolism of WDF isolates in PDB

Foams were detected within the broth of the WDF cultures from the 3 ^rd^ to the 18 ^th^ day of incubation at room temperature. Foaming of the cultures might be indicative of the production of gases by these WDF during growth in PDB; these gases could represent by-products of cellular metabolism. It has been reported that the transition from primary to secondary metabolism is marked by the depletion of nutrients in the culture medium ^19^. This study investigated the time point at which the dextrose was completely metabolized by the WDF during culturing in PDB; 2 out of the 54 WDF isolates (C9 and F7) were selected and used for this assay. The initial dextrose concentration in the freshly inoculated PDB was 550 mmol/L and decreased drastically to 40 mmol/L by the 3 ^rd^ day of incubation. Dextrose levels were not detected in the PDB on the 8 ^th^ day of incubation. This observation could suggest that transition to secondary metabolism occurred prior to the 8 ^th^ day of incubation of the fungal cultures and might represent the onset of production of antimicrobial compounds. Unprocessed results of dextrose metabolism assays, in addition to all other raw results, are available as *Underlying data*.

### Antimicrobial activities of extracts from WDF cultures

After 48 days of incubation of the 54 WDF cultures, it was observed that C7, E5, F2, F4 and F6 produced the highest yield of extract (200 mg) while the lowest yield (25 mg) was obtained from the WDF isolate designated B3. A significant number of the extracts were either yellowish or creamy in color.

**Figure 2.**
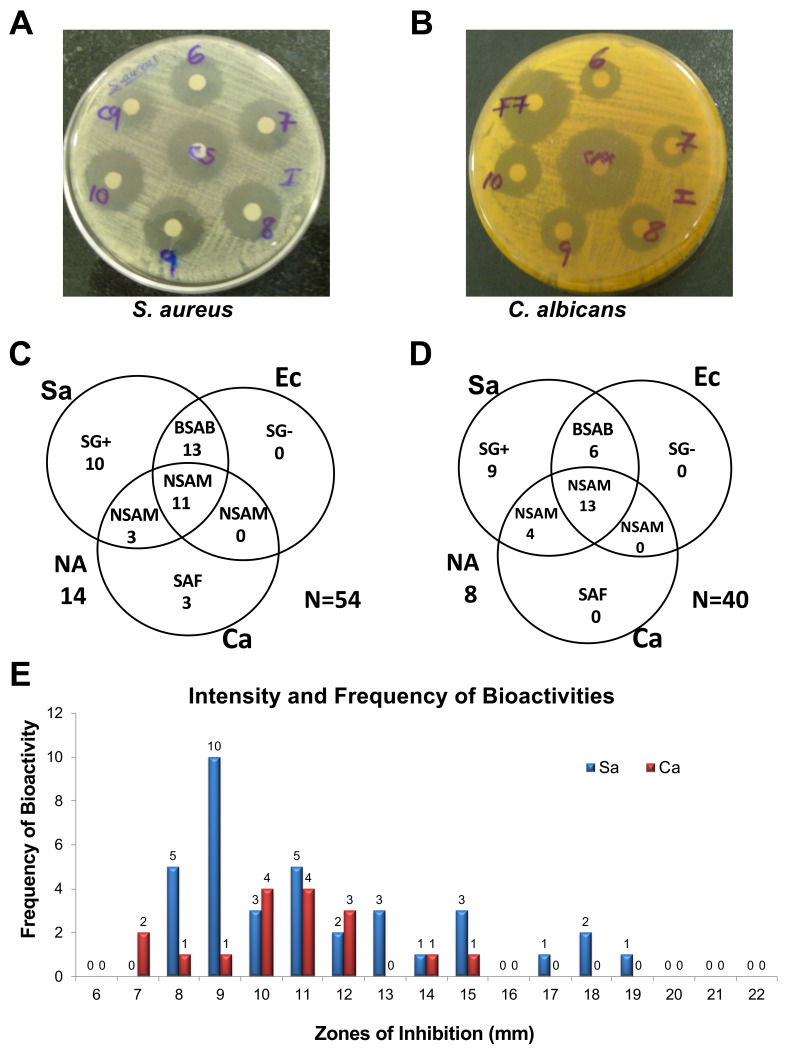
Primary and confirmatory screening for antimicrobial activities produced by WDF against *S. aureus*, *E. coli* and *C. albicans*. Examples of bioassay plates of wood-decaying fungi (WDF) extracts showing growth inhibition zones for *S. aureus* ( **A**) and *C. albicans* ( **B**) with streptomycin and cyclopirox olamine control antibiotics, respectively. Venn diagram showing the antimicrobial activities detected in the primary screen for 54 WDF extracts ( **C**) and confirmatory screen for 40 WDF extracts ( **D**). The frequency and potency of antimicrobial activity against *S. aureus* and *C. albicans* for the primary screening of the 54 WDF extracts ( **E**). NSAM, non-selective antimicrobial activity; BSAB, broad-spectrum antimicrobial; SG+, selective antibacterial activity against Gram-positive *S. aureus*; SAF, selective antifungal activity against *C. albicans*; SG-, selective antibacterial activity against Gram-negative *E. coli*.

The disc diffusion assay revealed that 40 out of the 54 extracts exhibited antimicrobial activity against at least one of the test organisms ( Figure 2A, B). Out of these 40 bioactive extracts, 11 extracts exhibited non-selective antimicrobial activity (NSAM) towards all the three test organisms, while 13 extracts showed antibacterial activity towards both *S. aureus* and *E. coli*. Additionally, 10 extracts exhibited selective antibacterial activity against only the gram-positive (SG+) *S. aureus*, while three other extracts showed selective antifungal activity (SAF) towards *C. albicans*. Notably, none of the 54 extracts exhibited selective antibacterial activity against only the gram-negative (SG-) *E. coli* ( Figure 2C).

The 40 WDF isolates that produced antimicrobial agents were re-cultured in PDB to validate the production of these bioactive metabolites. During re-culturing of these WDF isolates, foaming was detected from the 3 ^rd^ to the 21 ^st^ day of incubation. A total of 8 out of the 40 extracts from these WDF cultures did not retain their antimicrobial activity against neither of the three test organisms. Moreover, none of the 40 extracts exhibited selective antifungal activity against *C. albicans*. Despite these observations, 80% of the 40 extracts showed antimicrobial activity against at least one of the test organisms ( Figure 2D), thereby validating the production of antimicrobial compounds from these indigenous WDF. Interestingly, four of the extracts showed higher antibacterial activity against *S. aureus*, relative to streptomycin (10 μg) which had a zone of inhibition of 16 mm ( Figure 2E).

### Time course for production of antimicrobial compounds from WDF cultures

**Figure 3.**
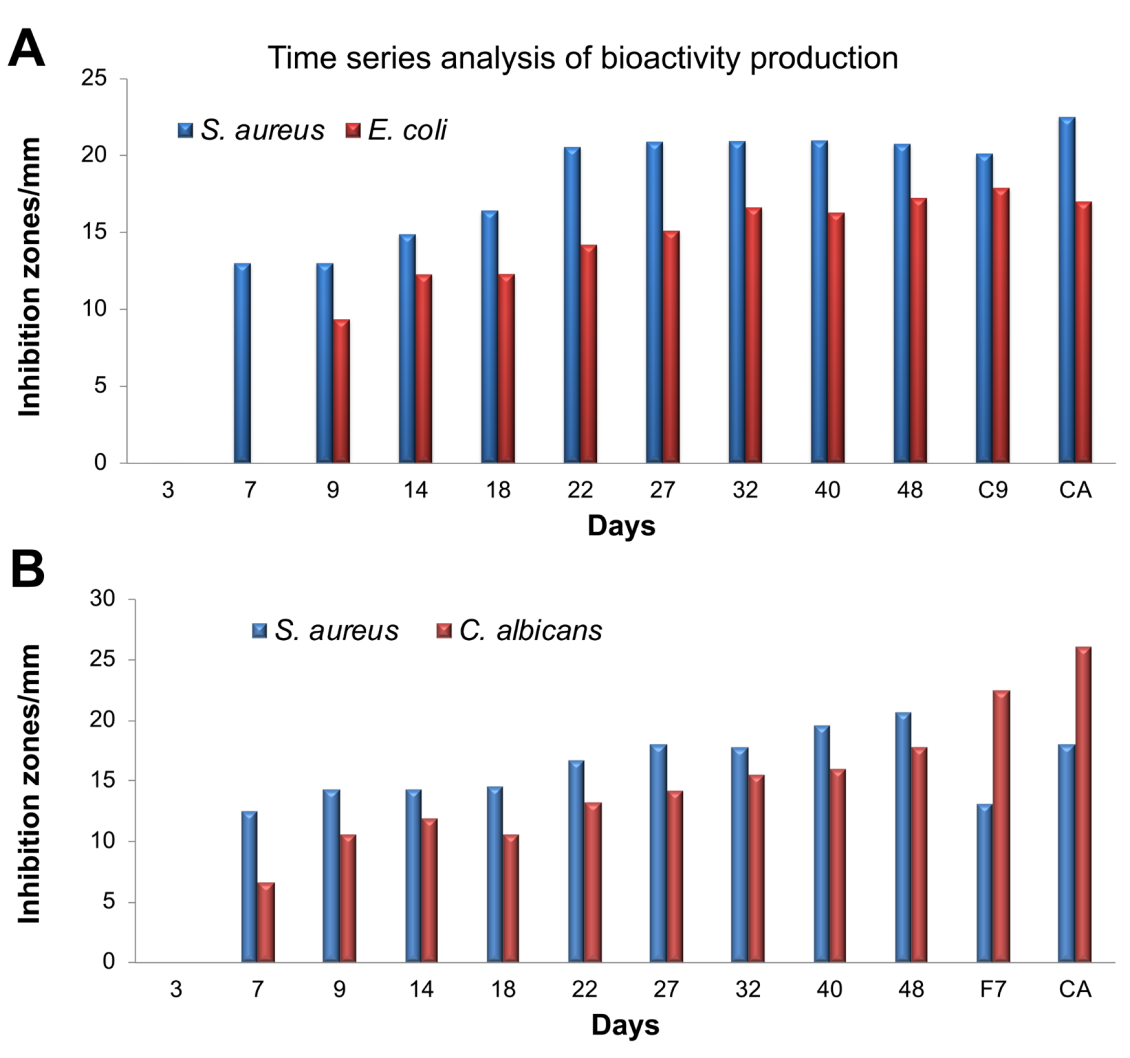
Time series analysis for the production of antimicrobial compounds by wood-decaying fungi. The WDF isolates C9 and F7 were used for the assay against *S. aureus* and *E. coli* ( **A**) and *S. aureus* and *C. albicans* ( **B**). Control antibiotics (CA): *S. aureus*, Streptomycin; *E. coli*, Kanamycin; *C. albicans*, Cyclopirox olamine. Antimicrobial activity of the extracts from the indicated time points was measured using the disc-diffusion assay, along with control antibiotics and reference samples of both C9 and E7.

Our earlier observation that WDF completely depleted dextrose from PDB by the 8 ^th^ day of incubation of the fungal cultures led us to hypothesize that these WDF began production of secondary metabolites by day 8. The two WDF isolates (C9 and F7) that were used for the dextrose clearance assay were chosen to investigate the time course of secondary metabolite production by the WDF cultures. TLC analysis of the extracts from the time course assay revealed no distinct and consistent band pattern. However, the disc diffusion assay showed that the C9 cultures produced antimicrobial compounds against *S. aureus* starting from the 7 ^th^ day and it attained maximum productivity of these compounds on the 22 ^nd^ day of culturing. These C9 extracts also exhibited antibacterial activity against *E. coli* from the 9 ^th^ day of culturing and maximum production of antimicrobial compounds was attained on day 32. In comparison, *S. aureus* was found to be more susceptible to extracts from the C9 cultures than *E. coli* over the entire sampling period ( Figure 3A).

Extracts from the F7 cultures exhibited antimicrobial activity against both *C. albicans* and *S. aureus* from the 7 ^th^ day and increased steadily until the 48 ^th^ day of culturing. The zones of inhibition observed for *S. aureus* were usually bigger than those for *C. albicans* ( Figure 3B). These observations indicate that the complete depletion of dextrose from the fungal cultures by day 8 is a good indicator of initiation of secondary metabolite production, including antimicrobial compounds.

### Fractionation of six WDF extracts exhibiting multiple antimicrobial activities

**Figure 4.**
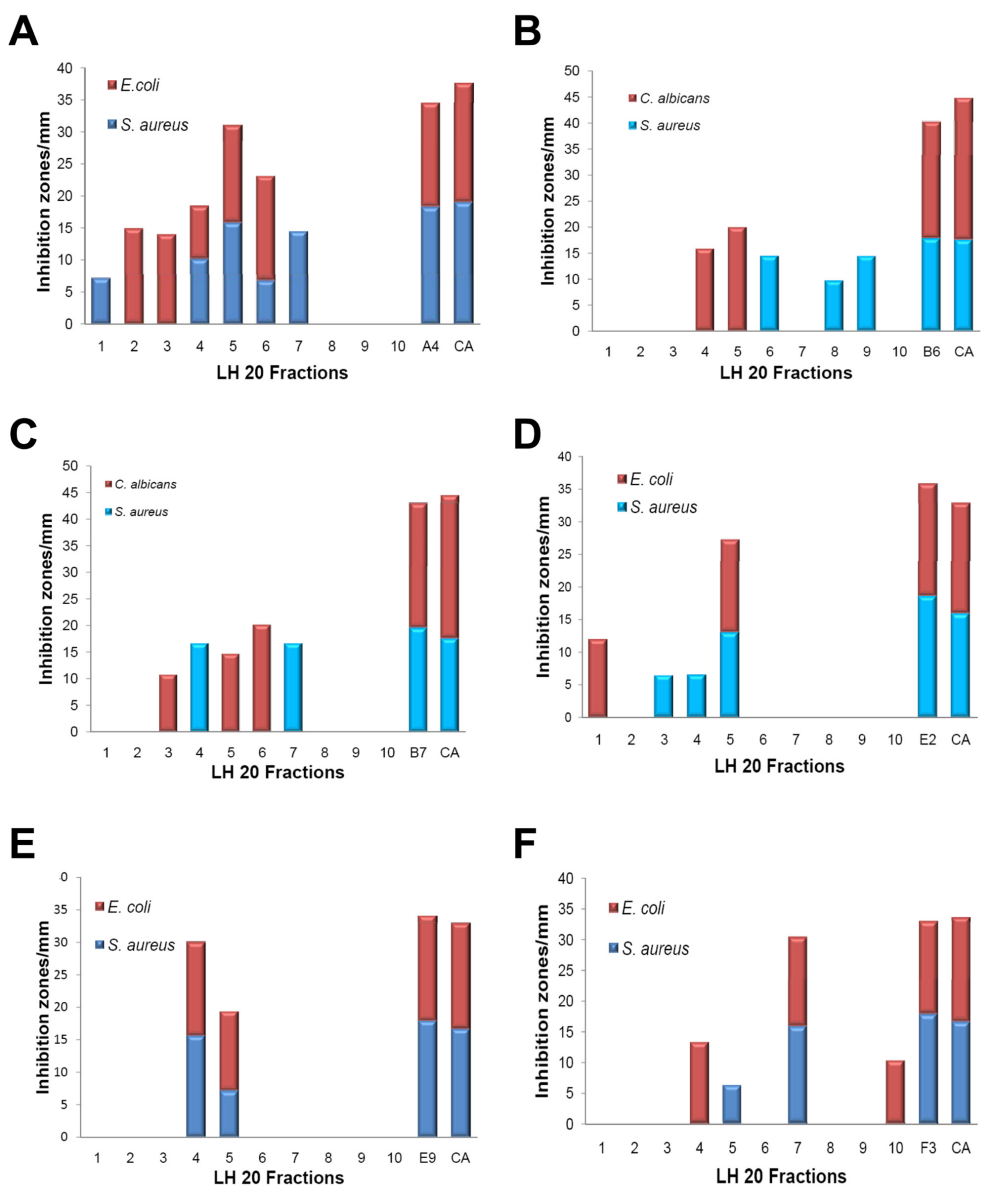
Analysis of the fractionated wood-decaying fungi (WDF) extracts exhibiting multiple antimicrobial activities. Prior to the bioassay, six selected WDF extracts (A4, B6, B7, E2, E9 and F3) were fractionated by sephadex LH-20 chromatography. The antimicrobial activities of the fractions obtained were measured using the disc diffusion assay with control antibiotics and original unfractionated extracts. The plot of zone of inhibitions for A4 ( **A**), B6 ( **B**), B7 ( **C**), E2 ( **D**), E9 ( **E**) and F3 ( **F**). Control antibiotics (CA): *S. aureus*, Streptomycin; *E. coli*, Kanamycin; *C. albicans*, Cyclopirox olamine.

Data from the antimicrobial assays revealed that a significant number of the WDF extracts exhibited antimicrobial activity against more than one of the three test organisms ( Figures 2C, D). Fractionation of six of these extracts (A4, B6, B7, E2, E9, F3) by sephadex LH-20 chromatography generated ten fractions for each extract. For the ten fractions obtained from the A4 extract, five fractions exhibited antimicrobial activity against *S. aureus*. The zones of inhibition that were recorded for two of these fractions (fractions 2 and 5; Figure 4A) were relatively high, suggesting that either a high amount of a single bioactive compound was present in these fractions or multiple antimicrobial compounds were present in each fraction. Similarly, five of the fractions from the A4 extract also exhibited antimicrobial activity against *E. coli*. Interestingly, three of these fractions (fractions 4, 5 and 6) exhibited broad-spectrum activity against both *S. aureus* and *E. coli* ( Figure 4A). Out of the ten fractions from the B6 extract, three fractions showed antibacterial activity against *S. aureus* while two other fractions exhibited antifungal activity against *C. albicans* ( Figure 4B).

For the B7 extract, antibacterial activity against *S. aureus* was detected in two out of the ten fractions whilst three other fractions showed antifungal activity against *C. albicans* ( Figure 4C). One out of the ten fractions from the E2 extract exhibited antibacterial activity against both *S. aureus* and *E. coli* ( Figure 4D). Selective antibacterial activity against *S. aureus* was detected in two of the fractions from the E2 extract whilst only one fraction was active against *E. coli* alone ( Figure 4D). Two out of the ten fractions from the E9 extract showed broad-spectrum antibacterial activity against *S. aureus* and *E. coli* ( Figure 4E). For the F3 extract, three out of the ten fractions showed selective antibacterial activity against *E. coli* while two fractions were active against *S. aureus* ( Figure 4F). Collectively, these observations may suggest that the broad-spectrum antimicrobial activities exhibited by the extracts selected for this assay, were predominantly due to the presence of different antimicrobial compounds in these extracts. Nonetheless, the fractions from these six extracts would require additional purification procedures in order to confirm the presence of different antimicrobial compounds within each of these extracts.

### Terpenes are the predominant compounds produced by the WDF isolates

**Figure 5.**
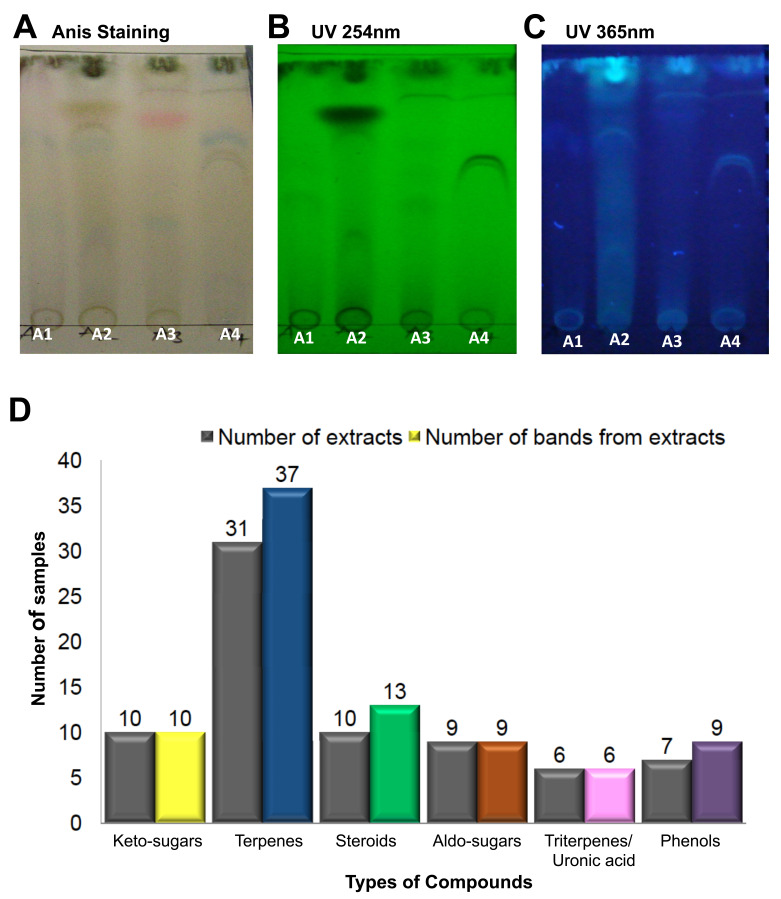
Diversity and distribution of compounds on the anisaldehyde-stained thin-layer chromatography (TLC) plates. The wood-decaying fungi (WDF) extracts A1, A2, A3 and A4 were run on TLC plates using the following solvent system as mobile phase; ethyl acetate: acetonitrile: petroleum ether (7:2:1). The TLC plates were visualized under UV and sprayed with anisaldehyde reagent in visible light. Examples of TLC plates showing the output of the various detection systems used in the analysis: ( **A**) Anisaldehyde staining ( **B**) UV 254 nm, ( **C**) UV 365 nm. ( **D**) The number of WDF extracts producing the indicated compounds (first bar) and the total number of individual colored bands produced by the WDF (second bar); the compound types are inferred from the corresponding colors.

TLC analysis was performed for all the 54 extracts in order to obtain preliminary insights on the diversity of compounds in the fungal extracts ( Figure 5). Each extract from the 54 WDF cultures produced several bands on the TLC plate, with different profiles and Rf values, which suggest that each of the WDF isolates were distinct from each other. After staining of TLC plates with anisaldehyde, bands corresponding to terpenes (blue bands) were observed in 31 out of the 54 WDF extracts; a few of these 31 extracts showed more than one distinct blue band. Thus, a total of 37 blue bands were detected from these 31 fungal extracts. Uronic acids (pink bands) and phenolic bands (purple bands) were found in 6 and 7 WDF extracts, respectively. Additionally, steroids (green bands) and keto-sugars (yellow bands) were detected in 10 WDF extracts, either exclusively or together with other compound types. Correlation analysis revealed that terpenes were present in most of the WDF extracts exhibiting antimicrobial activities. Hence, we infer that terpenes may contribute to the observed antimicrobial activity of the bioactive extracts.

## Discussion

This study explored the potential of indigenous WDF as sources of new antimicrobial compounds. A high proportion of these indigenous WDF exhibited either antibacterial or antifungal activities, demonstrating that these fungi are sources of antimicrobial compounds. Subsequent purification of the bioactive LH 20 fractions was not performed in this study to obtain the relevant antimicrobial compounds in their pure form. Nonetheless, it can be postulated that novel antimicrobial compounds can be obtained from these indigenous fungi since fungal secondary metabolites have been shown to be sources of novel bioactive compounds ^20– 22^. Moreover, the use of only a small collection of WDF isolates for this study indicates that the great biodiversity of WDF in Ghana represents potential sources of new compound structures which could be developed as novel drugs against microbial pathogens. The drug discovery programme initiated through this precursor study will conduct large scale fermentation to allow the exploitation of the vast diversity of bioactive compounds detected in this project.

Terpenes were the predominant compounds detected by TLC analysis of the extracts. The strong correlation between the occurrence of terpenes and antimicrobial activity might suggest a high probability of isolating terpenes as antimicrobial compounds. However, antibiotics exhibit antimicrobial activity at low concentrations, thus, it is possible the pharmacologically-active secondary metabolites in these extracts are non-terpenes and were undetected by TLC analysis due to the low abundance of these metabolites. Detection of other types of compounds in the bioactive WDF extracts could imply that the antimicrobial activity of the pharmacologically-active metabolites could either be dependent or boosted by the presence of these other compounds. Thus, identification of these active metabolites would provide a vital platform for studies on their synergy with the other classes of compounds detected in the extracts, such as steroids, glycosides and phenols. The synergistic studies would be useful for development of new antibiotic combination chemotherapy against both drug susceptible and multidrug resistant microbial pathogens.

The antimicrobial activity of the WDF extracts corroborates the relevance of fungal secondary metabolism to drug discovery. The preliminary data provided by this study also shows that the complete depletion of readily-available nutrients (such as dextrose) from the culture medium leads to initiation of fungal secondary metabolism. We recommend further studies that would investigate optimal conditions for culturing these indigenous WDF in order to boost immediate accumulation of antimicrobial agents following initiation of fungal secondary metabolism. Ultimately, it is intended that these efforts would contribute to efficient isolation of several new drug candidates to enhance the global fight against infectious diseases.

## Conclusion

This study has demonstrated that the great biodiversity of WDF in Ghana represents a valuable resource for discovery of antimicrobial agents, many of which might be novel to the drug discovery industries.

## Data availability

Open Science Framework: Wood Decaying Fungi (WDF) as Anti-infective Source Study. https://doi.org/10.17605/OSF.IO/H5GP9 ^19^.

The file “WDF and anti-infectives study Raw Data_Additional file.zip” contains the data underlying the results of this study, including antimicrobial activity at each time point, dextrose concentrations, zones of inhibition, images of plates and antimicrobial activity following repeated culture.

Data are available under the terms of the Creative Commons Zero “No rights reserved” data waiver (CC0 1.0 Public domain dedication).
