# Supplementary material for: Wood-decaying fungi found in Southern Ghana: A potential source of new anti-infective compounds
Source: AAS Open Res. 2019 Aug 27;2:20. Originally published 2019 Jun 7. [Version 2] doi: 10.12688/aasopenres.12957.2 (PMC9648367; doi:10.12688/aasopenres.12957.2)
Supplement: Peer review history

# Peer review: Reviewer response for version 2

**DOI:** 10.21956/aasopenres.14081.r27155

**Published:** 2019-09-11

**Authors:** Isaac Kingsley Amponsah, Francis Ackah Armah

**Affiliations:** 1 Department of Pharmacognosy, Faculty of Pharmacy & Pharmaceutical Sciences, College of Health Sciences, Kwame Nkrumah University of Science and Technology, Kumasi, Ghana; 2 Department of Biomedical Sciences, University of Cape Coast, Cape Coast, Ghana

I have noted the great job the authors have done in addressing the concerns raised in the first edition of the manuscript.

 The information flow is great and the method has been improved to afford repeatability.

Is the work clearly and accurately presented and does it cite the current literature?

Partly

If applicable, is the statistical analysis and its interpretation appropriate?

Not applicable

Are all the source data underlying the results available to ensure full reproducibility?

Yes

Is the study design appropriate and is the work technically sound?

Yes

Are the conclusions drawn adequately supported by the results?

Partly

Are sufficient details of methods and analysis provided to allow replication by others?

Partly

Reviewer Expertise:

Phytochemistry, structural elucidation, antimicrobial assays including anti- mycobacterium assays and antibiofil forming assays, standardisation of herbal products or medicinal plant monographs, Natural products chemistry

We confirm that we have read this submission and believe that we have an appropriate level of expertise to confirm that it is of an acceptable scientific standard.

# Peer review: Reviewer response for version 1

**DOI:** 10.21956/aasopenres.14036.r27021

**Published:** 2019-07-15

**Authors:** Isaac Kingsley Amponsah, Francis Ackah Armah

**Affiliations:** 1 Department of Pharmacognosy, Faculty of Pharmacy & Pharmaceutical Sciences, College of Health Sciences, Kwame Nkrumah University of Science and Technology, Kumasi, Ghana; 2 Department of Biomedical Sciences, University of Cape Coast, Cape Coast, Ghana

**Title**

 Wood decaying Fungus (WDF) samples were taken from **just three regions **of Ghana, I suggest authors revise the title to include ‘some’ so it reads “ **some** wood-decaying fungi found in Ghana:………”

**ABSTRACT**

**Method:** does not reflect the methods outlined in the manuscript. The first sentence is the objective of the study and thus does not belong to this section. Authors should revise this section to reflect what is in the manuscript.

**INTRODUCTION**

 To say that a high proportion of drugs in clinical use come from fungal sources is an emphatic statement. Therefore authors should provide the appropriate **reference.**

 Replace ‘ **contribute to’** in the last sentence of the introduction to **‘address’**. Novel antimicrobial agents will address global health concerns not contribute to global health concerns.

*Objectives of the research should be revised to accommodate the study of the metabolism and time course for the production secondary metabolites outlined elsewhere in this manuscript.*

**METHOD**

 This part was poorly written. Kindly revise to make the manuscript very comprehensible.

- For example to start this section with ‘images were acquired for each fungus……..’ does not allow the reader to follow the sequence of how the research was done. Kindly begin with the sample site and how they were collected
- Include details of the morphological characteristics which afforded the 54 WDF. Reference to relevant literature should be made to afford repeatability or independent verification of research.
- The rational for the TLC analysis and fractionation of the extracts was not clear. As revealed later in the results, not all 54 extracts were fractionated or TLC chromatographed. Authors must be clear with sufficient details of the method.
- From pages 7 & 8, it is clear different WDF isolate extracts afforded different number of bulked fractions (5 in some cases) but page 4 (under fractionation) predisposes us to erroneously believe that fractionation yielded 10 bulked fractions in all cases.

The method section should be revised to provide clarity on which WDF isolate were selected for fractionation and how many bulked fractions were obtained from those extracts. Again the information flow, logical approach is very important and should be taken into account.

**RESULTS**

- This section also needs extensive revision. The procedure for the study of the metabolism of WDF isolates as well as the time course for the production of secondary metabolites by WDF were not outlined in the method section. This was embedded in the results and should be clearly written out under methods.
- The use of anisaldehyde as a differential chromogenic reagent is not strongly supported, except where authors had small quantities of extracts or fractions, LC-MS would have being appropriate.

**DISCUSSION**

 To postulate that ‘novel antimicrobial agents can be obtained from indigenous WDF’ is not supported by results of the current research and should be revised. Fractions were not purified and characterised to afford novel compounds. Authors may however make reference to other studies where secondary metabolites from fungal isolates afforded novel antimicrobial compounds and by extension may want to postulate same.

Is the work clearly and accurately presented and does it cite the current literature?

Partly

If applicable, is the statistical analysis and its interpretation appropriate?

Not applicable

Are all the source data underlying the results available to ensure full reproducibility?

Yes

Is the study design appropriate and is the work technically sound?

Yes

Are the conclusions drawn adequately supported by the results?

Partly

Are sufficient details of methods and analysis provided to allow replication by others?

Partly

Reviewer Expertise:

Phytochemistry, structural elucidation, antimicrobial assays including anti- mycobacterium assays and antibiofil forming assays, standardisation of herbal products or medicinal plant monographs, Natural products chemistry

We confirm that we have read this submission and believe that we have an appropriate level of expertise to confirm that it is of an acceptable scientific standard, however we have significant reservations, as outlined above.

# Peer review: Reviewer response for version 1

**DOI:** 10.21956/aasopenres.14036.r27023

**Published:** 2019-07-15

**Authors:** Peter M. Njogu

**Affiliations:** 1 Department of Pharmaceutical Chemistry, School of Pharmacy, University of Nairobi, Nairobi, Kenya

The Kingdom Fungi has a rich heritage in the pharmaceutical industry as a source of numerous life-saving medicines in current clinical use, and this legacy will undoubtedly continue expanding. The contribution of fungi to the medical field is especially notable in the antimicrobial and anticancer therapeutic areas. The presently reported research work by the Group of Patrick Kobina Arthur is a confirmation that secondary metabolism in fungi will continue being a potential resource in antimicrobial drug discovery. 

 The background of the study is well presented and supported by relevant and current literature. The procedures are well described for reproducibility, except for two points:

- The incubation conditions of test microorganisms are reported as 37°C for 18 h. It is not clear whether this applies to the fungus *Candida albicans* as well.
- Some procedures are described in the Results section. Nevertheless, for majority of such instances, this is understandable given that decisions to progress in a particular direction of the research were based on prior laboratory work. However, the procedure for validation of antimicrobial activity of the 40 extracts could very well fit in the Methods section. 

Discussion section is well done, with the authors showing the relevance of their research work and results to the wider context of drug discovery and development in the near future. There are two points that may need clarity:

- The authors seem to infer that terpenes were likely the antimicrobially-active phytochemicals since they were the predominant class of compounds in the analyzed extracts. This may not necessarily be true. It is an established fact that pharmacologically-active natural compounds exist in very low concentrations and produce activity at even much lower concentrations. And this is the true definition of an *antibiotic*. The correct interpretation would be that terpenes were the predominant compounds and may contribute to the observed antimicrobial activity. 
- The authors have written: "It has previously been reported that the majority of bioactive compounds isolated from the fungus *Corynespora sp*. were polyketides". It is not clear to the reader why the authors are making reference to *Corynespora sp* at this juncture, given that it has not been specifically alluded to in the previous sections of the article. 

In summary, the research work is a great contribution to drug discovery and development of novel antimicrobial compounds from the African biodiversity.

Is the work clearly and accurately presented and does it cite the current literature?

Yes

If applicable, is the statistical analysis and its interpretation appropriate?

Partly

Are all the source data underlying the results available to ensure full reproducibility?

Yes

Is the study design appropriate and is the work technically sound?

Yes

Are the conclusions drawn adequately supported by the results?

Yes

Are sufficient details of methods and analysis provided to allow replication by others?

Yes

Reviewer Expertise:

Medicinal Chemistry; Pharmaceutical Research

I confirm that I have read this submission and believe that I have an appropriate level of expertise to confirm that it is of an acceptable scientific standard.
